# Case Report: A refractory unusual tetrad of overlap syndrome involving rheumatoid arthritis, Sjögren’s syndrome, autoimmune hepatitis, and type 1 renal tubular acidosis, successfully treated with a BLyS/APRIL dual inhibitor

**DOI:** 10.3389/fimmu.2025.1558059

**Published:** 2025-03-18

**Authors:** Wenjing Wang, Xin Ma, Bei Zhang, Zhibo Zhang, Xinfeng Wu, Hongwei Jiang, Xiaofei Shi

**Affiliations:** ^1^ Department of Rheumatology and Immunology, The First Affiliated Hospital of Henan University of Science and Technology, Henan, China; ^2^ Henan Key Laboratory of Rare Diseases, Endocrinology and Metabolism Center, The First Affiliated Hospital of Henan University of Science and Technology, Henan, China

**Keywords:** rheumatoid arthritis, Sjögren’s syndrome, autoimmune hepatitis, type 1 renal tubular acidosis, Telitacicept, BLyS/APRIL dual inhibitor

## Abstract

**Introduction:**

Rheumatoid arthritis (RA) and Sjögren’s syndrome (SS) are systemic autoimmune conditions. SS frequently occurs associated with RA. In patients with RA, those with SS exhibit a higher disease burden, increased disease activity, and more complex comorbidities compared with those without SS.

**Case report:**

We report a 54-year-old female patient who was previously diagnosed with early-stage RA less than 1 year ago. She was subsequently confirmed to have SS associated with RA. Additionally, she developed multiple autoimmune comorbidities, including autoimmune hepatitis and type 1 renal tubular acidosis. The patient resisted various treatments, including immunosuppressive drugs, disease-modifying antirheumatic drugs, and anti-inflammatory small-molecule drugs. This was evidenced by poor DA28 responses, persistent laboratory abnormalities, and ongoing symptoms and signs. Finally, she responded well to Telitacicept, a BLyS/APRIL dual inhibitor.

**Discussion:**

Even in the early stage, multiple autoimmune comorbidities can exhibit high levels of disease activity and may not respond to conventional therapies. Telitacicept, the first dual inhibitor of BLyS/APRIL, has the potential to provide significant efficacy and safety for RA patients who also have overlapping SS and other autoimmune diseases that do not respond to standard treatments. The limitations included the absence of a liver biopsy and the short follow-up period.

## Introduction

Rheumatoid arthritis (RA) is a systemic autoimmune inflammatory disease with distinguished autoantibody production and autoreactive T cells in the blood and synovial structures (1). Sjögren’s syndrome (SS) is a systemic autoimmune disease with a wide variety of presentations (2). SS frequently occurs with RA. The estimated prevalence rate for SS prevalence in patients with RA ranges from 4 to 31% (2, 3). Patients with RA and SS may experience a greater disease burden and higher levels of disease activity, and they may be associated with more systemic diseases than those with RA alone (4). Moreover, it remains unclear whether RA patients with SS will develop a higher likelihood of resistance to treatments.

## Case description

A 54-year-old woman with previously diagnosed RA less than 1year ago at another facility was admitted to our hospital due to worsening symptoms and a poor response to treatments. The patient had complaints of chronic swelling and migratory pain in multiple joints accompanied by morning stiffness for 1 year, along with numbness in her hands and feet for 8 months. Before being admitted to our hospital, she had poor response to ordinary inflammatory suppressors, including non-steroid anti-inflammatory drugs (NSAIDs), steroids, and immunosuppressors, alone or in combination. After 1 month of treatment with standard doses of celecoxib and leflunomide, her alanine transaminase (ALT) and aspartate transaminase (AST) levels were elevated. The status of her liver function before the treatments was unknown.

**Figure 1:**
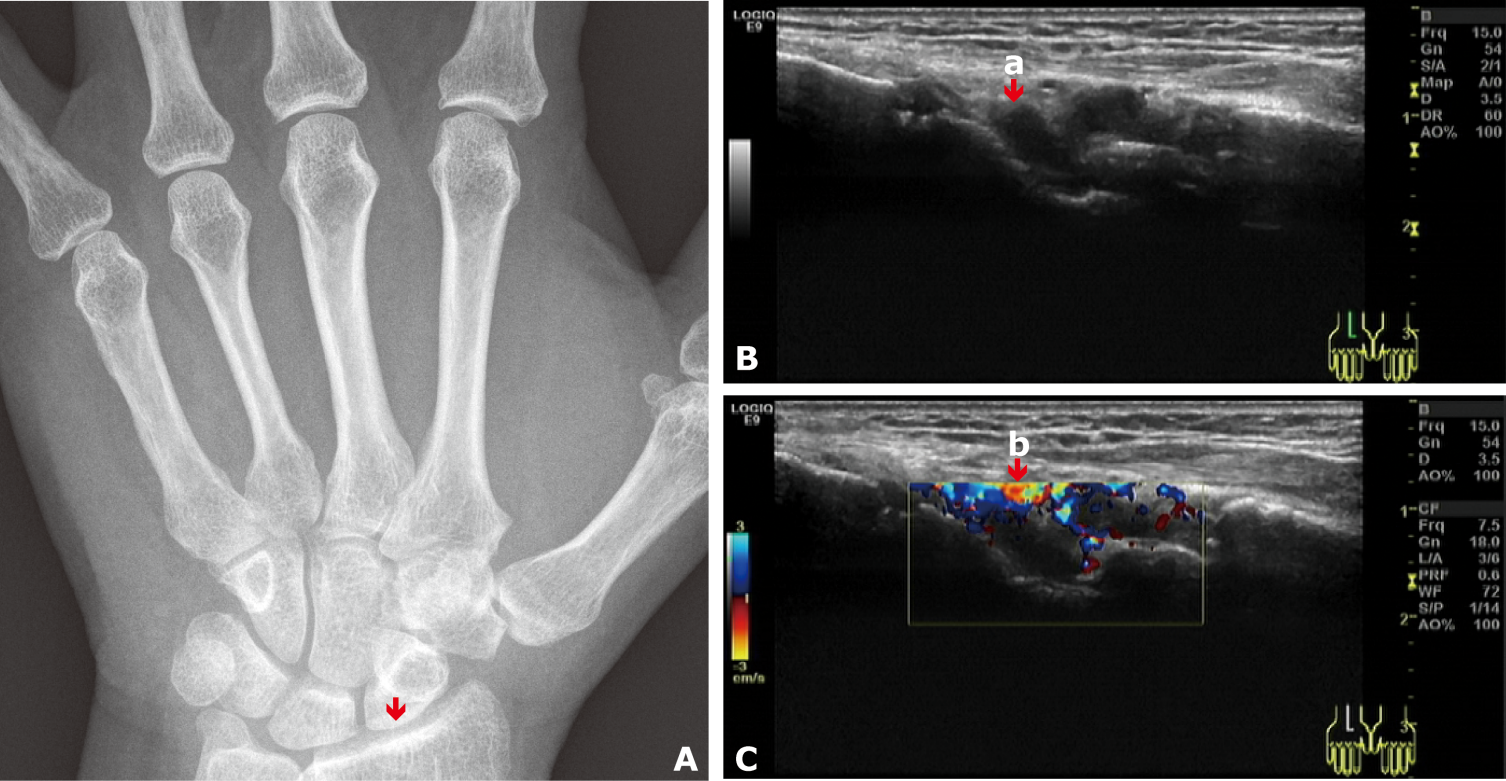
**(A)** DR image of the left wrist joint showed no signs of RA. The arrow indicates the left radiocarpal joint, where signs were observed on the Doppler scan. **(B)** Grayscale Doppler sagittal image of the left radiocarpal joint: The arrow (a) indicates synovial hypertrophy. **(C)** Color Doppler image of the left radiocarpal joint: The arrow (b) indicates hypervascularity.

Upon admission, her clinical and laboratory abnormalities met the diagnostic criteria for RA, achieving a point of seven according to the 2010 ACR/EULAR rheumatoid arthritis classification criteria (5): a) Joint involvement (2 points) refers to swollen and tender right 3rd to 4th proximal interphalangeal joints and left wrist joint on physical examination, without large joints involvement; b) serology (3 points) refers to positive rheumatoid factor (RF) (84 U/L, >3× upper limit normal [ULN]) and positive anti-cyclic citrullinated peptide antibody (ACCPA) (121 U/mL, >3× ULN); c) acute phase reactants (1 point) refers to abnormal C-reactive protein (CRP) (13.1 mg/L) and erythrocyte sedimentation rate (ESR) (30 mm/h); and d) duration of symptoms ≥6 weeks (1 point). She also had a positive anti-mutated citrullinated vimentin antibody (AMCVA), elevated immunoglobulin G (IgG) (18.2 g/L), and reduced complement 4 (C4) (0.41 g/L). No signs of RA were observed in the digital radiography (DR) images of both hands, aligning with the early manifestations of RA (**Figure 1A**). The sonograph evaluation revealed synovial hypertrophy and hypervascularity in the left radiocarpal joint, demonstrating active synovitis (**Figures 1B, C**).

**Figure 2:**
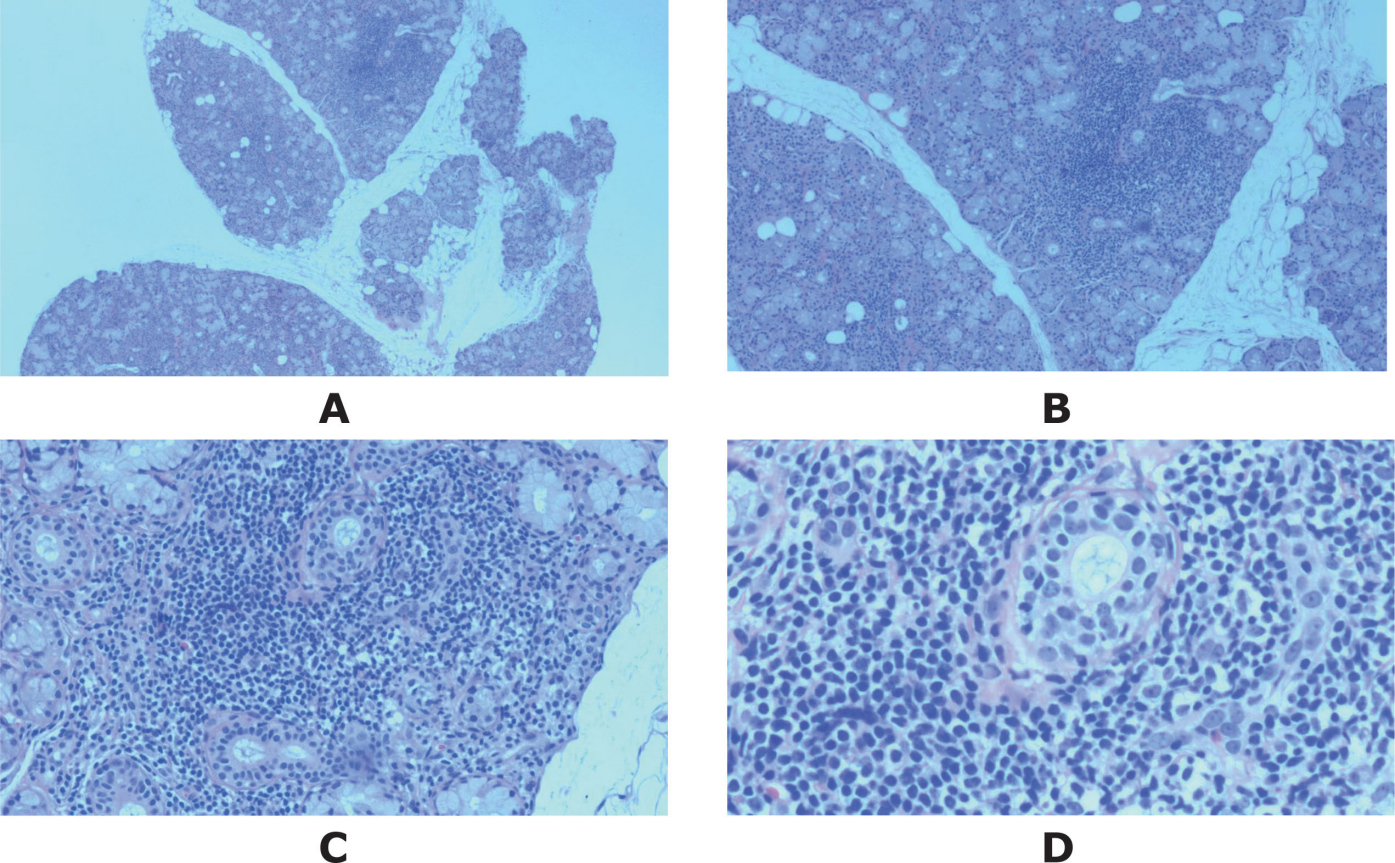
The microphotograph of the LGS biopsy, stained with hematoxylin and eosin, showed one focus within the whole glandular area containing more than 50 lymphocytes and plasmacytes. Original magnification: **(A)** ×2, **(B)** ×4, **(C)** ×10, **(D)** ×20.

More specifically, she experienced two sudden episodes of flaccid quadriparesis during her tours, which were clinically diagnosed as hypokalemic periodic paralysis in primary healthcare units without serum potassium testing, and the symptoms were relieved after potassium supplementation. Due to the features of multiple autoimmune comorbidities in patients with overlapping RA and SS, the possibilities of SS and distal (type 1) renal tubular acidosis (dRTA) had to be considered. The patient had positive ANA-granular type (1:100), anti-SSA/Ro 60KD, anti-SSA/Ro 52KD, anti-SSB/La antibodies, and anti-smooth muscle antibody (ASMA) (1:100). The labial salivary gland (LSG) pathology revealed that lymphocytes and plasma cells infiltrated the interstitium, maintaining a normal gland lobular structure, with a focus score of one according to the EULAR guideline (6) (**Figure 2**). Ophthalmic examination revealed positive corneal staining (+), abnormal Schirmer test (right eye 5 mm/5 min, left eye 7 mm/5 min), and abnormal tear film break-up time (right eye 6 s, left eye 7 s). The diagnosis of SS was established according to the 2016 American College of Rheumatology/European League Against Rheumatism Classification Criteria for Primary Sjögren’s Syndrome, achieving a score of eight (7).

No instances of flaccid quadriparesis were observed during her visits to our hospital. However, she needed to continue taking potassium citrate to prevent feelings of weakness. Her serum potassium levels were generally abnormal, with two results indicating hypokalemia (3.30, 2.50 mmol/L) and one result at the lower limit of normal (3.50 mmol/L). Her serum calcium consistently indicated hypocalcemia (0.99, 0.84, 0.22 mmol/L). Her serum HCO_3_^−^ levels ranged from 20.5 to 27.4 mmol/L, whereas the serum chloride and sodium levels remained normal. The results of the serum anion gap remained within the normal range. Her urine PH was 7.5, significantly higher than 5.3 (8). Her glomerular filtration function was normal. As a result, the diagnosis of dRTA secondary to SS can be established (8). Autoimmune diseases, such as Sjogren’s syndrome (SS), have been considered the most common cause of dRTA (9).

Her ALT and AST levels remained abnormal after the dosage of leflunomide was reduced and withdrawn, and viral hepatitis was ruled out by serologic testing. The diagnosis of autoimmune hepatitis (AIH) was established according to the simplified criteria for the diagnosis of autoimmune hepatitis by the International Autoimmune Hepatitis Group (IAIHG) (10) with a score of 6: a) ANA 1:100 ≥ 1:80 (2 scores); b) IgG 18.12 g/L ≥ 1.1 × UNL (2 scores); c) absence of viral hepatitis (2 scores). However, the patient declined to undergo histological testing of the liver.

After being diagnosed with RA at another hospital, the patient began treatment with celecoxib and leflunomide (20 mg once daily). One month later, her ALT/AST levels elevated and remained abnormal even after reducing the leflunomide dosage and ultimately withdrawing it. The therapy regimen was then changed to methotrexate (7.5 mg once a week) and methylprednisolone (methyl-pred, 6 mg once daily). She stopped taking methyl-pred herself shortly after. Her pain and swelling in the joints did not improve significantly. Upon her admission to our hospital, she started treatment with hydroxychloroquine (HCQ) and iguratimod, but her response to the regimen was still poor. She then received a new regimen of baricitinib 2 mg once daily, HCQ 0.2 g twice daily, and total glucosides of paeony (TGP) 0.6 g three times daily for 2 months. Two more months later, she continued to experience persistent swelling and pain in joints, along with elevated levels of IgG (18.12 g/L) and RF (202 IU/mL). She subsequently underwent additional treatment that involved stem cell infusion. Two months later, her symptoms relapsed, presenting as pain in both knee joints and then spreading to multiple joints in her hands and feet without any noticeable swelling. She was readmitted to our hospital due to numbness and convulsions of both hands. Her laboratory abnormalities included an ESR of 23 mm/h, CRP of 13.1 mg/dL, RF of 169 IU/mL, ACCPA of 104.3 U/mL, ANA of 228 AU/mL, and complement C3 of 0.76 g/L. The measurement of disease activity indicated moderate RA activity with a DAS28(ESR) score of 4.86, a DAS(CRP) score of 4.45 scores, a Clinical Disease Activity Index (CDAI) score of 21, and low-level SS activity with an EULAR Sjogren’s syndrome disease activity index (ESSDAI) score (11) of 4, and a Sjögren’s Syndrome Patient Reported Index (ESSPRI) score (12) of 3.4. Due to her poor response to the prior treatments, Telitacicept was administered at a dosage of 160 mg once a week in combination with hydroxychloroquine 0.2 g twice daily. The laboratory abnormalities disappeared after 3 months of treatment with the new regimen. The only exception was the level of ANA, which fluctuated between 200 and 260 AU/mL. Her response to the therapy remained stable throughout a half-year follow-up period. The re-evaluation of disease activity showed that the RA was in clinical remission, with a DAS28(ESR) score of 2.22, a DAS(CRP) score of 1.5, and a CDAI score of 5.5. The SS also achieved clinical remission, with an ESSDAI score of 2 and an ESSPRI score of 3.1.

**Figure 3:**
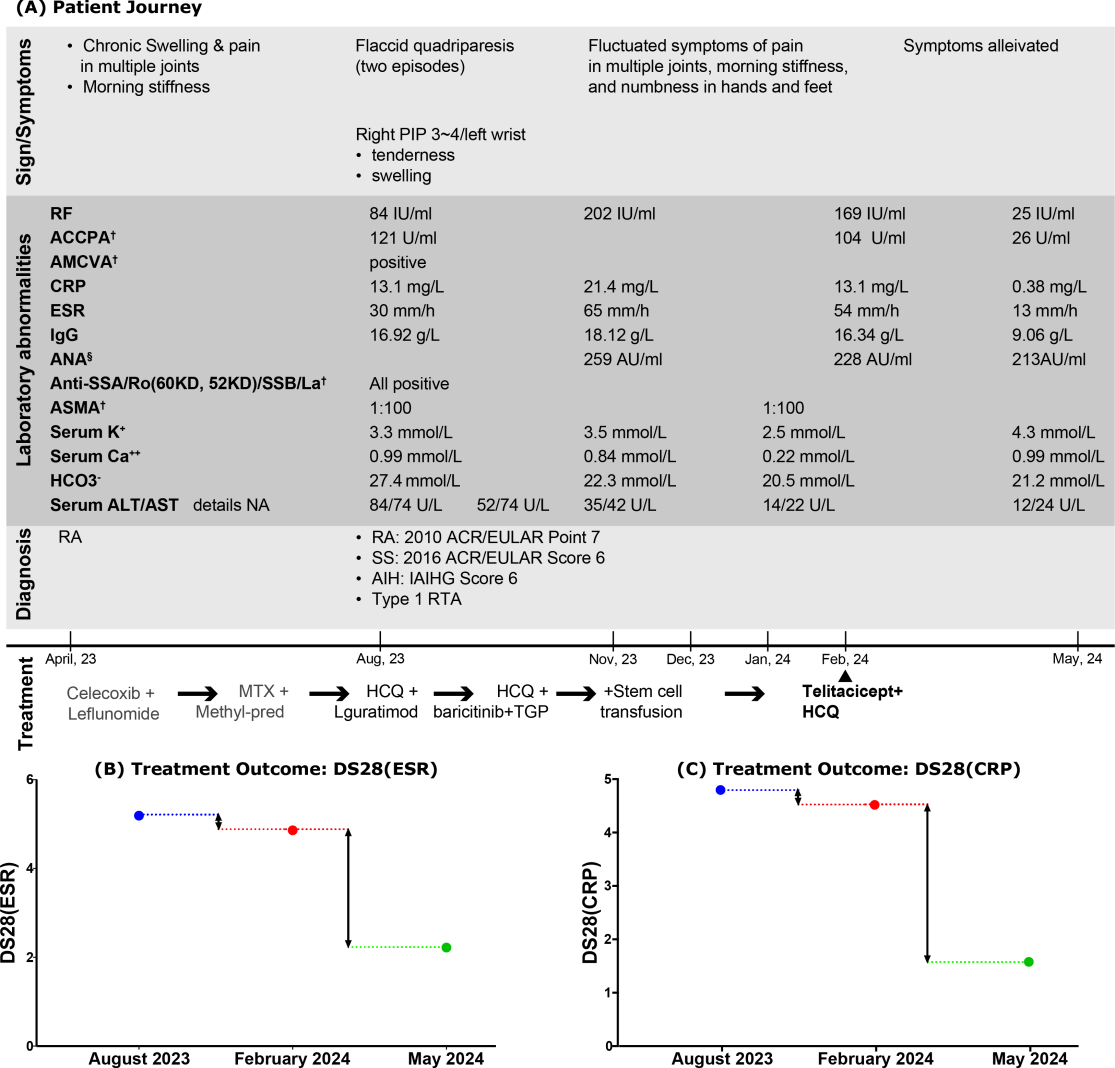
Comprehensive events: **(A)** related to the Patient Journey; **(B, C)** focused on Treatment Outcomes. PIP, proximal interphalangeal joint; RF, rheumatoid factor; ACCPA, anti-cyclic citrullinated peptide antibody; AMCVA, anti-mutated citrullinated vimentin antibody; ASMA, anti-smooth muscle antibody; CRP, C-reaction protein; ESR, erythrocyte sedimentation rate; ANA, antinuclear antibody; Anti-SSA/Ro, anti-Sjögren’s-syndrome type A antibodies; Anti-SSB/La, anti-Sjögren’s syndrome type B antibodies; ASMA, anti-smooth muscle antibody; HCO_3_^−^, bicarbonate; ALT, serum alanine transaminase; AST, serum aspartate transaminase; DS28(ESR), disease activity score in RA by assessing joints and ESR; DS28(CRP), disease activity score in RA by assessing joints and CRP; HCQ, hydroxychloroquine; TGP, total glucosides of paeony. ^†^The detections were performed using indirect immunofluorescence. ^§^ANA was quantified using chemiluminescent immunoassay.

The patient journey, along with laboratory abnormalities, diagnoses, and treatment courses, is summarized in **Figure 3**.

## Discussion

SS often occurs alongside other autoimmune diseases, including RA (2). The term “SS associated with RA” is now preferred over “SS secondary to RA” because it emphasizes that these autoimmune conditions coexist rather than one being secondary to the other (13). SS may be a marker of a more aggressive joint disease in patients with RA (14). The presence of SS adds to the RA disease burden, negatively impacts patients’ daily lives, and is associated with increased autoimmune comorbidities (14). Limited studies have identified molecular mechanisms related to RA with SS, which may serve as potential treatment targets (15). This reported case exhibited an unusual overlap of four autoimmune conditions, accompanied by numerous autoantibodies, and resisted multiple therapies. Her refractoriness to immunosuppressive drugs, disease-modifying antirheumatic drugs, and anti-inflammatory small-molecule drugs were manifested as poor DA28(ESR) and DA28(CRP) responses, persistent laboratory abnormalities including elevated levels of autoantibodies, RF, CRP, ESR, and IgG, along with ongoing symptoms and signs. She finally reached durable remission from autoimmune conditions after receiving treatment with Telitacicept in combination with hydroxychloroquine. It has been reported that SS patients with a higher focus score are associated with extraglandular involvement that requires multiple medications (16). However, this reported case did not exhibit a high focus score. Different profiles of anti-Ro antibodies were significantly associated with clinical phenotypic features in connective tissue diseases. Compared with patients with isolated anti-Ro52 or anti-Ro60 antibodies, the patients with combined anti-Ro52 and anti-Ro60 antibodies are more likely to suffer from xerophthalmia and xerostomia. In contrast, among the patients with isolated anti-Ro52 antibodies, idiopathic inflammatory myopathy and systemic lupus erythematosus were identified as the most common diagnoses. Hypocomplementemia, hyperglobulinemia, and proteinuria were reported particularly common in patients with anti-Ro60 antibodies (17). Although the patient refused a liver biopsy, preventing histopathological evidence, AIH has been clinically diagnosed in this patient after excluding drug-induced liver injury and viral hepatitis. Reports indicate that the prevalence of AIH is 1.4%~35% in SS and 1.6%~5.4% in RA (18). Recent new insights suggested that the level of ANA might not always accurately reflect the severity of an autoimmune disease and can fluctuate even with treatment (19). This reported case’s ANA level also varied in an abnormal range after treatment with Telitacicept. Heavy disease burden in patients with overlapping autoimmune conditions may lead to resistance to therapies. Recent studies indicate that B-cell-targeted therapy can be effective in patients with severe and refractory systemic diseases (20). Telitacicept is the first BLyS/APRIL dual inhibitor. It simultaneously prevents B cells from differentiating into plasma cells by blocking the B lymphocyte stimulator (BLyS) and reduces the secretion of autoantibodies by plasma cells by inhibiting a proliferation-inducing ligand (APRIL) (21). It provides a more comprehensive inhibition of B-cell development and activation. A phase 3 randomized, double-blind trial (NCT03016013) presented at the 2023 ACR Annual Meeting showed that Telitacicept was effective and well tolerated in the treatment of moderate to severe RA patients with inadequate response to methotrexate (22). A randomized, placebo-controlled clinical trial demonstrated that Telitacicept resulted in a significant DAS 28 response in patients with moderate to severe RA activity (23).

In conclusion, RA associated with multiple autoimmune comorbidities may exhibit increased disease activity and resistance to treatment. Telitacicept, as the first BLyS/APRIL dual inhibitor, may demonstrate significant efficacy and safety in patients with this clinical condition who resist conventional therapies. The limitations of this study included the absence of a liver biopsy to pathologically prove the diagnosis of AIH and the short follow-up period. Additionally, due to its retrospective nature, more response predictors of clinical responses, such as the count of CD27(+) B cells and the level of IgM-RF, were not available.

## Data Availability

The original contributions presented in the study are included in the article/supplementary material. Further inquiries can be directed to the corresponding author.
